# *catena*-Poly[diammonium [diaqua­bis(pyridine-2,4-dicarboxyl­ato-κ^2^ *N*,*O* ^2^)cuprate(II)] [[diaqua­copper(II)]-μ-pyridine-2,4-dicarboxyl­ato-κ^3^ *N*,*O* ^2^:*O* ^2′^-[tetra­aqua­cadmium(II)]-μ-pyridine-2,4-dicarboxyl­ato-κ^3^ *O* ^2^:*N*,*O* ^2′^] hexa­hydrate]

**DOI:** 10.1107/S1600536809046911

**Published:** 2009-11-14

**Authors:** Guan-Hua Wang, Zhi-Gang Li, Heng-Qing Jia, Ning-Hai Hu, Jing-Wei Xu

**Affiliations:** a The State Key Laboratory of Electroanalytical Chemistry, Changchun Institute of Applied Chemistry, Chinese Academy of Sciences, Changchun 130022, People’s Republic of China; b Graduate School, Chinese Academy of Sciences, Beijing 100039, People’s Republic of China

## Abstract

The title mixed-metal complex, {(NH_4_)_2_[Cu(C_7_H_3_NO_4_)_2_(H_2_O)_2_][CdCu(C_7_H_3_NO_4_)_2_(H_2_O)_6_]·6H_2_O}_*n*_, contains one octa­hedrally coordinated Cd^II^ center and two octa­hedrally coordinated Cu^II^ centers, each lying on an inversion center. The two Cu^II^ atoms are each coordinated by two O atoms and two N atoms from two 2,4-pydc (2,4-H_2_pydc = pyridine-2,4-dicarboxylic acid) ligands in the equatorial plane and two water mol­ecules at the axial sites, thus producing two crystallographically independent [Cu(2,4-pydc)_2_(H_2_O)_2_]^2−^ metalloligands. One metalloligand exists as a discrete anion and the other connects the Cd(H_2_O)_4_ units, forming a neutral chain. O—H⋯O and N—H⋯O hydrogen bonds connects the polymeric chains, complex anions, ammonium cations and uncoordinated water mol­ecules into a three-dimensional supra­molecular network.

## Related literature

For general background to coordination polymers, see: Caneschi *et al.* (2001 ▶); Dong *et al.* (2000 ▶); Evans & Lin (2002 ▶); Kitagawa *et al.* (1999 ▶, 2004 ▶, 2006 ▶). For related structures, see: Li *et al.* (2008 ▶); Noro *et al.* (2002*a* ▶,*b* ▶); Wang *et al.* (2009 ▶).
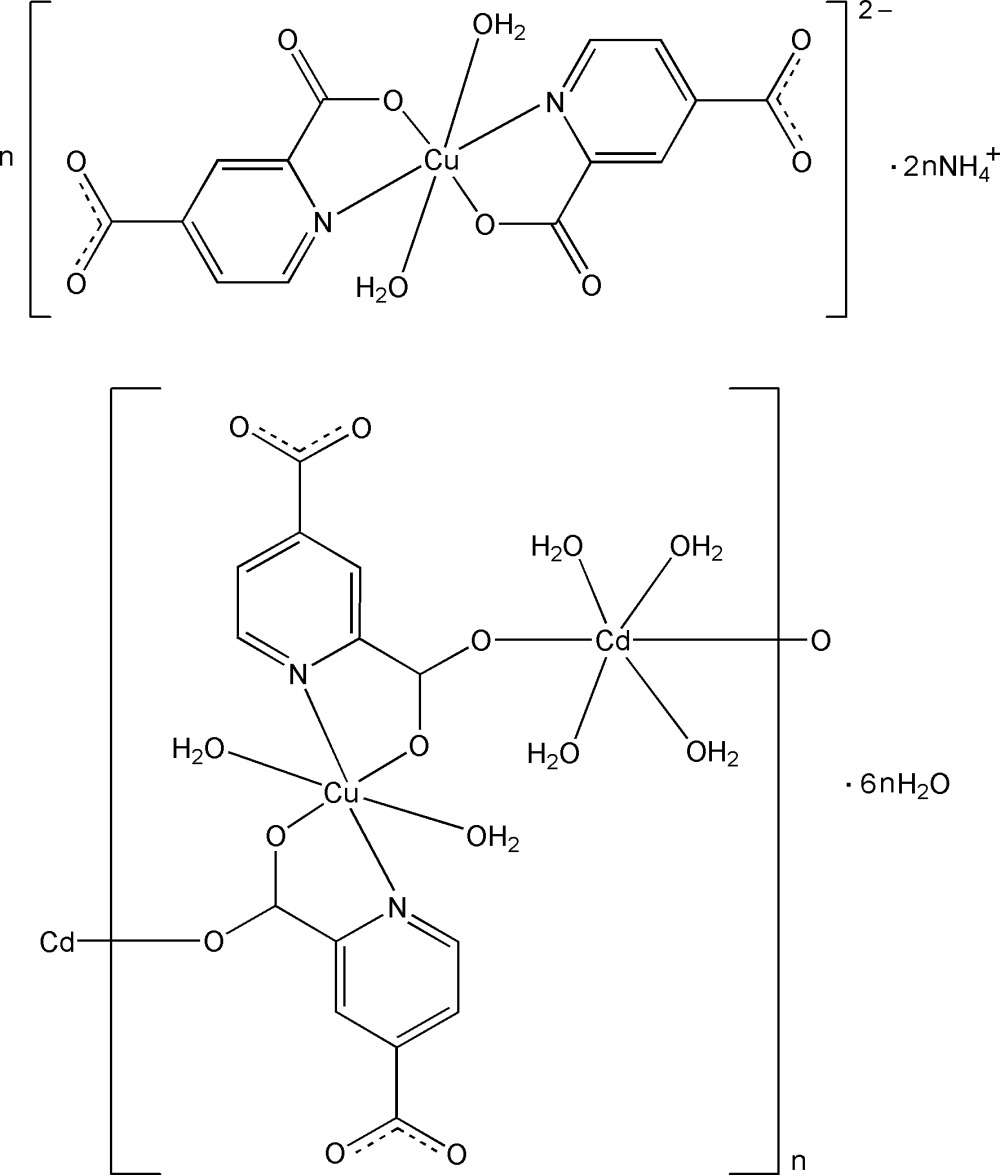


## Experimental

### 

#### Crystal data

(NH_4_)_2_[Cu(C_7_H_3_NO_4_)_2_(H_2_O)_2_][CdCu(C_7_H_3_NO_4_)_2_(H_2_O)_6_]·6H_2_O
*M* *_r_* = 1188.20
Triclinic, 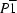

*a* = 10.4520 (19) Å
*b* = 10.5252 (19) Å
*c* = 10.6733 (19) Å
α = 102.869 (2)°
β = 103.536 (2)°
γ = 94.834 (2)°
*V* = 1101.3 (3) Å^3^
*Z* = 1
Mo *K*α radiation
μ = 1.54 mm^−1^
*T* = 293 K
0.22 × 0.20 × 0.16 mm

#### Data collection

Bruker SMART APEX CCD diffractometer
Absorption correction: multi-scan (*SADABS*; Sheldrick, 1996 ▶) *T*_min_ = 0.720, *T*_max_ = 0.785
6205 measured reflections
4212 independent reflections
3869 reflections with *I* > 2σ(*I*)
*R*_int_ = 0.011

#### Refinement

*R*[*F* ^2^ > 2σ(*F* ^2^)] = 0.026
*wR*(*F* ^2^) = 0.073
*S* = 1.05
4212 reflections
361 parameters
31 restraints
H atoms treated by a mixture of independent and constrained refinement
Δρ_max_ = 0.67 e Å^−3^
Δρ_min_ = −0.41 e Å^−3^

### 

Data collection: *SMART* (Bruker, 2007 ▶); cell refinement: *SAINT* (Bruker, 2007 ▶); data reduction: *SAINT*; program(s) used to solve structure: *SHELXS97* (Sheldrick, 2008 ▶); program(s) used to refine structure: *SHELXL97* (Sheldrick, 2008 ▶); molecular graphics: *SHELXTL* (Sheldrick, 2008 ▶); software used to prepare material for publication: *SHELXTL*.

## Supplementary Material

Crystal structure: contains datablocks I, global. DOI: 10.1107/S1600536809046911/bg2303sup1.cif

Structure factors: contains datablocks I. DOI: 10.1107/S1600536809046911/bg2303Isup2.hkl

Additional supplementary materials:  crystallographic information; 3D view; checkCIF report

## Figures and Tables

**Table 1:** Hydrogen-bond geometry (Å, °)

*D*—H⋯*A*	*D*—H	H⋯*A*	*D*⋯*A*	*D*—H⋯*A*
O1*W*—H1*A*⋯O7^i^	0.94 (2)	1.80 (1)	2.739 (2)	171 (2)
O1*W*—H1*B*⋯O4^ii^	0.95 (2)	1.82 (1)	2.769 (2)	177 (3)
O2*W*—H2*A*⋯O3^ii^	0.95 (1)	1.72 (1)	2.657 (3)	168 (2)
O2*W*—H2*B*⋯O8^iii^	0.95 (1)	1.77 (1)	2.722 (2)	177 (2)
O3*W*—H3*A*⋯O6*W* ^iv^	0.94 (1)	1.84 (1)	2.781 (3)	172 (3)
O3*W*—H3*B*⋯O7^iv^	0.94 (1)	1.84 (1)	2.776 (3)	172 (3)
O4*W*—H4*A*⋯O3*W* ^v^	0.95 (1)	1.89 (1)	2.827 (3)	169 (2)
O4*W*—H4*B*⋯O4^i^	0.95 (1)	1.80 (1)	2.752 (3)	176 (2)
O5*W*—H5*A*⋯O4*W* ^vi^	0.95 (1)	2.10 (1)	3.048 (3)	170 (3)
O5*W*—H5*B*⋯O3	0.96 (1)	1.93 (1)	2.882 (3)	171 (3)
O6*W*—H6*A*⋯O6	0.95 (1)	1.79 (1)	2.742 (3)	173 (3)
O6*W*—H6*B*⋯O2*W* ^vii^	0.96 (3)	2.14 (2)	2.991 (3)	147 (2)
O7*W*—H7*A*⋯O2	0.95 (3)	2.09 (3)	3.009 (3)	163 (3)
O7*W*—H7*B*⋯O5*W*	0.95 (3)	1.93 (3)	2.861 (3)	165 (4)
N3—H31⋯O7*W* ^viii^	0.98 (2)	1.90 (2)	2.867 (3)	174 (2)
N3—H32⋯O8	0.99 (2)	1.92 (1)	2.886 (3)	164 (2)
N3—H33⋯O5*W* ^ix^	0.99 (1)	2.53 (2)	3.208 (4)	126 (2)
N3—H33⋯O5^x^	0.99 (1)	2.30 (2)	3.131 (3)	140 (2)
N3—H33⋯O6^x^	0.99 (1)	2.19 (2)	2.888 (3)	126 (2)
N3—H34⋯O8^xi^	0.99 (2)	2.34 (1)	3.277 (3)	157 (2)

Symmetry codes: (i) 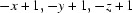; (ii) 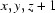; (iii) 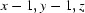; (iv) 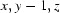; (v) 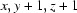; (vi) 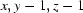; (vii) 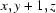; (viii) 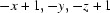; (ix) 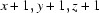; (x) 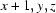; (xi) 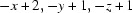.

**Fig. 1.**
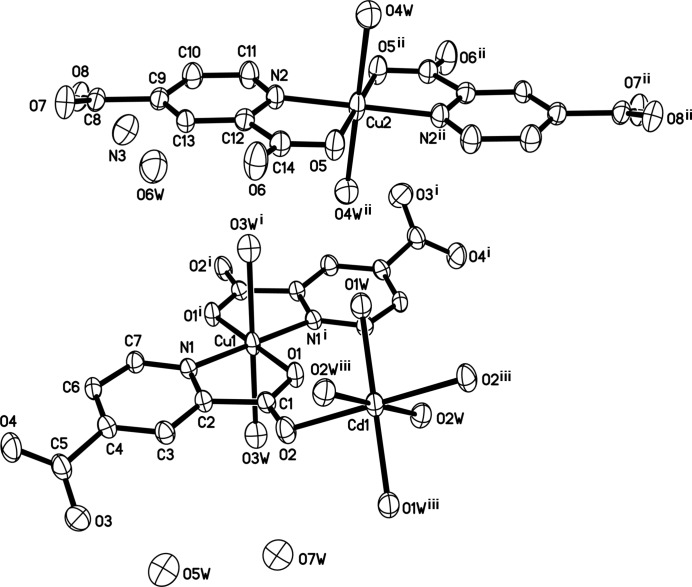
The asymmetric unit of the title compound, together with symmetry-related atoms to complete the Cd1, Cu1 and Cu2 coordination. Displacement ellipsoids are drawn at the 50% probability level. H atoms have been omitted for clarity. [Symmetry codes: (i) 1 - x, -y, 1 - z; (ii) 1 - x, 1 - y, 2 - z; (iii) -x, -y, 1 - z.]

**Fig. 2.**
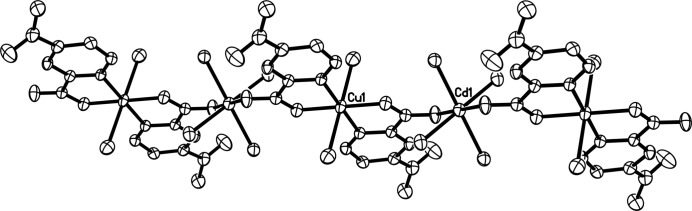
The one-dimensional chain in the title compound. H atoms have been omitted for clarity.

**Fig. 3.**
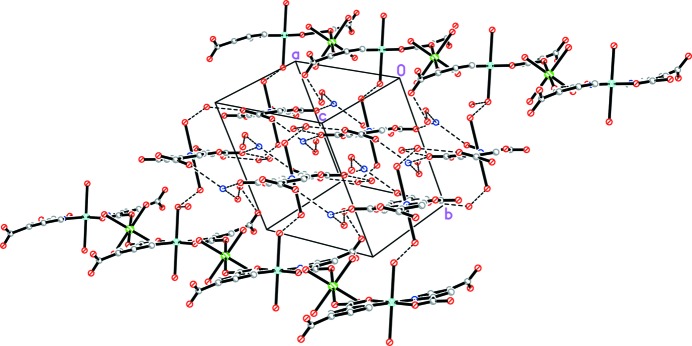
The crystal packing of the title compound. Dashed lines denote hydrogen bonds.

## supplementary crystallographic information

### Comment

Coordination polymers constructed from metal ions and bridging ligands have been of great interest owing to their structural diversities and fascinating properties (Caneschi *et al.*, 2001; Evans & Lin, 2002; Kitagawa *et al.*, 1999, 2004). In recent years, the design and synthesis of mixed-metal coordination polymers have received much attention because such heterometallic materials might exhibit interesting physical properties, resulting from interactions between two neighboring metal centers connected by a suitable linker (Dong *et al.*, 2000; Kitagawa *et al.*, 2006). Noro *et al.* (2002*a*, b) have prepared mixed-metal coordination polymers by using the Et_3_NH salt of a metalloligand, [Cu(2,4-pydc)_2_]^2-^ (2,4-H_2_pydc = pyridine-2,4-dicarboxylic acid). We prepared recently a mixed-metal complex with a metalloligand [Cu(2,5-pydc)_2_]^2-^ by a simplified synthetic method (Wang *et al.*, 2009). As a continuation of our work, we report here the synthesis and structure of the title compound.

The asymmetric unit of the title compound contains one six-coordinated Cd^II^ atom and two six-coordinated Cu^II^ atoms, each lying on an inversion center, two 2,4-pydc ligands, one ammonium ion, four coordinated water molecules and three uncoordinated water molecules (Fig. 1). Both Cu1 and Cu2 atoms have an axially elongated octahedral coordination geometry, defined by two O atoms and two N atoms from two 2,4-pydc ligands in the equatorial plane and two water molecules at the axial sites, thus producing two crystallographically independent [Cu(2,4-pydc)_2_(H_2_O)_2_]^2-^ metalloligands. In each metalloligand, the equatorial plane consists of *trans* N donors and *trans* O donors. The Cd^II^ ions coordinated by four water molecules are linked by the Cu1-metalloligands, *via* the bidentate-bridging 2-carboxylate groups, into a one-dimensional polymeric chain along the [100] direction (Fig. 2). The shortest Cu···Cd distance is 5.226 (1) Å. The 2,4-pydc ligand binds Cu1 and Cd1 atoms in a µ_2_-(κ^3^*N*,*O*^2^:*O*^2'^) mode with the 4-carboxylate group uncoordinated (Li *et al.*, 2008). The Cu2-metalloligand acts as a discrete divalent anion and does not interact with a second metal ion. The 2,4-pydc ligand in the Cu2-metalloligand adopts a (κ^2^*N*,*O*^2^) chelating mode with the 4-carboxylate group remaining idle. Extensive O—H···O and N—H···O hydrogen bonds (Table 1) assemble the various components into a supramolecular network (Fig. 3).

### Experimental

An aqueous solution (20 ml) of Cu(NO_3_)_2_.3H_2_O (0.125 g, 0.3 mmol) and a suspension of 2,4-H_2_pydc (0.083 g, 0.3 mmol) in ethanol (10 ml) were mixed and refluxed for 24 h until a clear solution was obtained. To this solution, an aqueous solution (5 ml) of CdCl_2_ (0.055 g, 0.5 mmol) was added. Aqueous NH_3_ (25%, 0.06 ml) was then slowly added to the reaction mixture. The resulting solution was filtered off. Blue block crystals were obtained by allowing the filtrate to stand at room temperature for several days.

### Refinement

H atoms on C atoms were positioned geometrically and refined using a riding model, with C—H = 0.93 Å and with *U*_iso_(H) = 1.2*U*_eq_(C). H atoms of water molecules and ammonium ion were located in a difference Fourier map and refined with distance restraints of O—H = 0.96 (1), H···H = 1.56 (1) Å, and N—H = 0.99 (1), H···H = 1.62 (1) Å, and with *U*_iso_(H) = 1.2*U*_eq_(O,*N*).

### Crystal data

**Table:**

(NH_4_)_2_[Cu(C_7_H_3_NO_4_)_2_(H_2_O)_2_][CdCu(C_7_H_3_NO_4_)_2_(H_2_O)_6_]·6H_2_O	*Z* = 1
*M**_r_* = 1188.20	*F*(000) = 604
Triclinic, *P*1	*D*_x_ = 1.792 Mg m^−^^3^
Hall symbol: -P 1	Mo *K*α radiation, λ = 0.71073 Å
*a* = 10.4520 (19) Å	Cell parameters from 4140 reflections
*b* = 10.5252 (19) Å	θ = 2.5–26.1°
*c* = 10.6733 (19) Å	µ = 1.54 mm^−^^1^
α = 102.869 (2)°	*T* = 293 K
β = 103.536 (2)°	Block, blue
γ = 94.834 (2)°	0.22 × 0.20 × 0.16 mm
*V* = 1101.3 (3) Å^3^	

### Data collection

**Table:**

Bruker SMART APEX CCD diffractometer	4212 independent reflections
Radiation source: fine-focus sealed tube	3869 reflections with *I* > 2σ(*I*)
graphite	*R*_int_ = 0.011
φ and ω scans	θ_max_ = 26.1°, θ_min_ = 2.0°
Absorption correction: multi-scan (*SADABS*; Sheldrick, 1996)	*h* = −12→8
*T*_min_ = 0.720, *T*_max_ = 0.785	*k* = −12→12
6205 measured reflections	*l* = −11→13

### Refinement

**Table:**

Refinement on *F*^2^	Primary atom site location: structure-invariant direct methods
Least-squares matrix: full	Secondary atom site location: difference Fourier map
*R*[*F*^2^ > 2σ(*F*^2^)] = 0.026	Hydrogen site location: inferred from neighbouring sites
*wR*(*F*^2^) = 0.073	H atoms treated by a mixture of independent and constrained refinement
*S* = 1.05	*w* = 1/[σ^2^(*F*_o_^2^) + (0.0412*P*)^2^ + 0.7186*P*] where *P* = (*F*_o_^2^ + 2*F*_c_^2^)/3
4212 reflections	(Δ/σ)_max_ = 0.004
361 parameters	Δρ_max_ = 0.67 e Å^−^^3^
31 restraints	Δρ_min_ = −0.41 e Å^−^^3^

### Fractional atomic coordinates and isotropic or equivalent isotropic displacement parameters (Å^2^)

**Table:**

	*x*	*y*	*z*	*U*_iso_*/*U*_eq_	
Cd1	0.0000	0.0000	0.5000	0.02604 (8)	
Cu1	0.5000	0.0000	0.5000	0.02460 (10)	
Cu2	0.5000	0.5000	1.0000	0.03268 (11)	
N1	0.44406 (17)	0.04440 (17)	0.32615 (17)	0.0221 (4)	
N2	0.60608 (19)	0.56353 (19)	0.88835 (18)	0.0282 (4)	
O1	0.30942 (15)	−0.02363 (16)	0.48585 (15)	0.0281 (3)	
O2	0.11497 (15)	−0.03483 (18)	0.34073 (16)	0.0342 (4)	
O3	0.1713 (2)	−0.0467 (2)	−0.13119 (19)	0.0591 (6)	
O4	0.30140 (19)	0.1332 (2)	−0.12763 (17)	0.0439 (5)	
O5	0.35050 (16)	0.49779 (19)	0.84955 (17)	0.0369 (4)	
O6	0.30210 (18)	0.5639 (2)	0.6633 (2)	0.0490 (5)	
O7	0.71230 (17)	0.72200 (19)	0.51106 (18)	0.0391 (4)	
O8	0.89977 (16)	0.65182 (18)	0.59992 (18)	0.0366 (4)	
C1	0.2382 (2)	−0.0169 (2)	0.3746 (2)	0.0239 (4)	
C2	0.3115 (2)	0.0180 (2)	0.2780 (2)	0.0227 (4)	
C3	0.2475 (2)	0.0220 (2)	0.1505 (2)	0.0269 (5)	
H3	0.1557	0.0001	0.1186	0.032*	
C4	0.3247 (2)	0.0598 (2)	0.0707 (2)	0.0267 (5)	
C5	0.2599 (2)	0.0495 (3)	−0.0752 (2)	0.0327 (5)	
C6	0.4603 (2)	0.0970 (2)	0.1247 (2)	0.0262 (5)	
H6	0.5124	0.1301	0.0763	0.031*	
C7	0.5176 (2)	0.0845 (2)	0.2518 (2)	0.0255 (4)	
H7	0.6094	0.1046	0.2858	0.031*	
C8	0.7820 (2)	0.6710 (2)	0.5938 (2)	0.0280 (5)	
C9	0.7192 (2)	0.6332 (2)	0.6975 (2)	0.0270 (5)	
C10	0.7977 (2)	0.6251 (3)	0.8193 (2)	0.0337 (5)	
H10	0.8898	0.6421	0.8379	0.040*	
C11	0.7380 (2)	0.5914 (3)	0.9128 (2)	0.0340 (5)	
H11	0.7913	0.5882	0.9948	0.041*	
C12	0.5292 (2)	0.5726 (2)	0.7720 (2)	0.0261 (5)	
C13	0.5820 (2)	0.6079 (2)	0.6751 (2)	0.0258 (4)	
H13	0.5262	0.6147	0.5958	0.031*	
C14	0.3820 (2)	0.5433 (2)	0.7580 (2)	0.0305 (5)	
O1W	0.14804 (16)	0.17674 (17)	0.64065 (16)	0.0324 (4)	
H1A	0.202 (2)	0.205 (3)	0.590 (2)	0.039*	
H1B	0.201 (2)	0.159 (3)	0.7184 (16)	0.039*	
O2W	0.08911 (16)	−0.15033 (17)	0.60774 (17)	0.0323 (4)	
H2A	0.121 (2)	−0.102 (2)	0.6985 (13)	0.039*	
H2B	0.023 (2)	−0.2214 (18)	0.603 (2)	0.039*	
O3W	0.46396 (19)	−0.24026 (19)	0.37308 (18)	0.0397 (4)	
H3A	0.3913 (16)	−0.275 (3)	0.399 (3)	0.048*	
H3B	0.5449 (13)	−0.261 (3)	0.419 (3)	0.048*	
O4W	0.4544 (2)	0.7280 (2)	1.10086 (19)	0.0445 (4)	
H4A	0.447 (3)	0.731 (3)	1.1887 (15)	0.053*	
H4B	0.5379 (17)	0.775 (3)	1.106 (3)	0.053*	
O5W	0.1659 (2)	−0.2927 (2)	−0.0551 (2)	0.0585 (6)	
H5A	0.2575 (14)	−0.294 (3)	−0.016 (3)	0.070*	
H5B	0.158 (3)	−0.213 (2)	−0.084 (3)	0.070*	
O6W	0.2430 (2)	0.6784 (2)	0.4538 (2)	0.0459 (5)	
H6A	0.260 (3)	0.632 (2)	0.522 (2)	0.055*	
H6B	0.210 (3)	0.7573 (18)	0.488 (3)	0.055*	
O7W	0.0227 (3)	−0.2976 (3)	0.1422 (3)	0.0668 (7)	
H7A	0.033 (3)	−0.2138 (19)	0.202 (3)	0.080*	
H7B	0.083 (3)	−0.297 (3)	0.088 (3)	0.080*	
N3	1.0503 (2)	0.4588 (2)	0.6944 (3)	0.0482 (6)	
H31	1.020 (2)	0.406 (2)	0.749 (2)	0.058*	
H32	0.9847 (18)	0.5165 (19)	0.666 (2)	0.058*	
H33	1.1357 (14)	0.5156 (19)	0.745 (2)	0.058*	
H34	1.065 (2)	0.4011 (19)	0.6132 (15)	0.058*	

### Atomic displacement parameters (Å^2^)

**Table:**

	*U*^11^	*U*^22^	*U*^33^	*U*^12^	*U*^13^	*U*^23^
Cd1	0.02452 (13)	0.03503 (14)	0.02213 (13)	0.00417 (9)	0.00922 (9)	0.01115 (9)
Cu1	0.02026 (19)	0.0393 (2)	0.01908 (19)	0.00659 (15)	0.00640 (15)	0.01509 (16)
Cu2	0.0253 (2)	0.0519 (3)	0.0262 (2)	−0.00001 (18)	0.00792 (17)	0.02147 (19)
N1	0.0217 (9)	0.0281 (9)	0.0180 (8)	0.0046 (7)	0.0060 (7)	0.0077 (7)
N2	0.0284 (10)	0.0354 (10)	0.0239 (9)	0.0021 (8)	0.0081 (8)	0.0132 (8)
O1	0.0248 (8)	0.0418 (9)	0.0230 (8)	0.0056 (7)	0.0091 (6)	0.0158 (7)
O2	0.0214 (8)	0.0579 (11)	0.0241 (8)	0.0019 (7)	0.0082 (7)	0.0113 (8)
O3	0.0686 (15)	0.0667 (14)	0.0275 (10)	−0.0263 (12)	−0.0090 (10)	0.0183 (9)
O4	0.0468 (11)	0.0582 (12)	0.0280 (9)	−0.0027 (9)	0.0038 (8)	0.0235 (9)
O5	0.0264 (9)	0.0565 (11)	0.0323 (9)	−0.0003 (8)	0.0089 (7)	0.0214 (8)
O6	0.0295 (9)	0.0816 (15)	0.0422 (11)	0.0023 (9)	0.0037 (8)	0.0367 (11)
O7	0.0360 (9)	0.0528 (11)	0.0423 (10)	0.0131 (8)	0.0178 (8)	0.0298 (9)
O8	0.0295 (9)	0.0450 (10)	0.0460 (10)	0.0076 (7)	0.0177 (8)	0.0241 (8)
C1	0.0238 (11)	0.0291 (11)	0.0201 (10)	0.0040 (8)	0.0076 (8)	0.0068 (8)
C2	0.0219 (10)	0.0292 (11)	0.0199 (10)	0.0055 (8)	0.0077 (8)	0.0087 (8)
C3	0.0220 (11)	0.0375 (12)	0.0216 (11)	0.0040 (9)	0.0047 (9)	0.0096 (9)
C4	0.0305 (12)	0.0319 (12)	0.0191 (10)	0.0054 (9)	0.0063 (9)	0.0089 (9)
C5	0.0345 (13)	0.0440 (14)	0.0214 (11)	0.0053 (11)	0.0069 (10)	0.0124 (10)
C6	0.0290 (11)	0.0314 (11)	0.0213 (10)	0.0021 (9)	0.0110 (9)	0.0092 (9)
C7	0.0231 (11)	0.0322 (12)	0.0236 (11)	0.0030 (9)	0.0088 (9)	0.0095 (9)
C8	0.0308 (12)	0.0278 (11)	0.0310 (12)	0.0035 (9)	0.0139 (10)	0.0129 (9)
C9	0.0305 (12)	0.0263 (11)	0.0285 (11)	0.0041 (9)	0.0127 (9)	0.0104 (9)
C10	0.0257 (12)	0.0453 (14)	0.0328 (12)	0.0026 (10)	0.0090 (10)	0.0149 (11)
C11	0.0263 (12)	0.0498 (15)	0.0291 (12)	0.0031 (10)	0.0058 (10)	0.0190 (11)
C12	0.0277 (11)	0.0267 (11)	0.0263 (11)	0.0037 (9)	0.0092 (9)	0.0091 (9)
C13	0.0280 (11)	0.0299 (11)	0.0223 (10)	0.0035 (9)	0.0085 (9)	0.0104 (9)
C14	0.0280 (12)	0.0377 (13)	0.0274 (12)	0.0017 (10)	0.0076 (10)	0.0123 (10)
O1W	0.0271 (8)	0.0448 (10)	0.0260 (8)	−0.0001 (7)	0.0065 (7)	0.0126 (7)
O2W	0.0289 (9)	0.0393 (9)	0.0310 (9)	0.0019 (7)	0.0074 (7)	0.0150 (7)
O3W	0.0398 (10)	0.0460 (11)	0.0390 (10)	0.0101 (8)	0.0139 (8)	0.0168 (8)
O4W	0.0488 (12)	0.0475 (11)	0.0367 (10)	−0.0018 (9)	0.0118 (9)	0.0120 (9)
O5W	0.0555 (13)	0.0568 (13)	0.0681 (15)	0.0083 (11)	0.0205 (12)	0.0208 (11)
O6W	0.0532 (12)	0.0450 (11)	0.0466 (11)	0.0098 (9)	0.0174 (9)	0.0205 (9)
O7W	0.0744 (17)	0.0650 (15)	0.0564 (15)	−0.0103 (13)	0.0218 (13)	0.0085 (12)
N3	0.0302 (12)	0.0545 (15)	0.0682 (17)	0.0102 (10)	0.0157 (11)	0.0277 (13)

### Geometric parameters (Å, °)

**Table:**

Cd1—O2	2.2850 (16)	C3—H3	0.9300
Cd1—O2^i^	2.2850 (16)	C4—C6	1.384 (3)
Cd1—O1W	2.3004 (17)	C4—C5	1.521 (3)
Cd1—O2W^i^	2.2915 (17)	C6—C7	1.389 (3)
Cd1—O2W	2.2915 (17)	C6—H6	0.9300
Cd1—O1W^i^	2.3004 (17)	C7—H7	0.9300
Cu1—O1^ii^	1.9523 (15)	C8—C9	1.519 (3)
Cu1—O1	1.9523 (15)	C9—C13	1.390 (3)
Cu1—N1	1.9819 (17)	C9—C10	1.389 (3)
Cu1—N1^ii^	1.9819 (17)	C10—C11	1.386 (3)
Cu1—O3W^ii^	2.539 (2)	C10—H10	0.9300
Cu1—O3W	2.539 (2)	C11—H11	0.9300
Cu2—O5	1.9553 (17)	C12—C13	1.385 (3)
Cu2—O5^iii^	1.9553 (17)	C12—C14	1.509 (3)
Cu2—N2	1.9862 (18)	C13—H13	0.9300
Cu2—N2^iii^	1.9862 (18)	O1W—H1A	0.94 (2)
Cu2—O4W^iii^	2.535 (2)	O1W—H1B	0.95 (2)
Cu2—O4W	2.535 (2)	O2W—H2A	0.95 (1)
N1—C7	1.334 (3)	O2W—H2B	0.95 (1)
N1—C2	1.342 (3)	O3W—H3A	0.94 (1)
N2—C11	1.336 (3)	O3W—H3B	0.94 (1)
N2—C12	1.340 (3)	O4W—H4A	0.95 (1)
O1—C1	1.266 (3)	O4W—H4B	0.95 (1)
O2—C1	1.239 (3)	O5W—H5A	0.95 (1)
O3—C5	1.246 (3)	O5W—H5B	0.96 (1)
O4—C5	1.244 (3)	O6W—H6A	0.95 (1)
O5—C14	1.275 (3)	O6W—H6B	0.96 (3)
O6—C14	1.224 (3)	O7W—H7A	0.95 (3)
O7—C8	1.255 (3)	O7W—H7B	0.95 (3)
O8—C8	1.253 (3)	N3—H31	0.98 (2)
C1—C2	1.509 (3)	N3—H32	0.99 (2)
C2—C3	1.379 (3)	N3—H33	0.99 (1)
C3—C4	1.397 (3)	N3—H34	0.99 (2)
			
O2—Cd1—O2^i^	180.00 (6)	N1—C2—C3	122.80 (19)
O2—Cd1—O2W^i^	84.25 (6)	N1—C2—C1	114.40 (18)
O2^i^—Cd1—O2W^i^	95.75 (6)	C3—C2—C1	122.80 (19)
O2—Cd1—O2W	95.75 (6)	C2—C3—C4	118.1 (2)
O2^i^—Cd1—O2W	84.25 (6)	C2—C3—H3	121.0
O2W^i^—Cd1—O2W	180.0	C4—C3—H3	121.0
O2—Cd1—O1W	95.40 (6)	C6—C4—C3	118.78 (19)
O2^i^—Cd1—O1W	84.60 (6)	C6—C4—C5	121.7 (2)
O2W^i^—Cd1—O1W	85.58 (6)	C3—C4—C5	119.4 (2)
O2W—Cd1—O1W	94.42 (6)	O4—C5—O3	126.5 (2)
O2—Cd1—O1W^i^	84.60 (6)	O4—C5—C4	118.3 (2)
O2^i^—Cd1—O1W^i^	95.40 (6)	O3—C5—C4	115.1 (2)
O2W^i^—Cd1—O1W^i^	94.43 (6)	C4—C6—C7	119.3 (2)
O2W—Cd1—O1W^i^	85.57 (6)	C4—C6—H6	120.3
O1W—Cd1—O1W^i^	179.999 (1)	C7—C6—H6	120.3
O1^ii^—Cu1—O1	180.0	N1—C7—C6	121.5 (2)
O1^ii^—Cu1—N1	96.26 (7)	N1—C7—H7	119.2
O1—Cu1—N1	83.74 (7)	C6—C7—H7	119.2
O1^ii^—Cu1—N1^ii^	83.74 (7)	O8—C8—O7	125.5 (2)
O1—Cu1—N1^ii^	96.26 (7)	O8—C8—C9	117.5 (2)
N1—Cu1—N1^ii^	180.0	O7—C8—C9	117.0 (2)
O1^ii^—Cu1—O3W^ii^	85.99 (6)	C13—C9—C10	117.8 (2)
O1—Cu1—O3W^ii^	94.01 (6)	C13—C9—C8	121.3 (2)
N1—Cu1—O3W^ii^	92.15 (7)	C10—C9—C8	120.8 (2)
N1^ii^—Cu1—O3W^ii^	87.85 (7)	C11—C10—C9	119.8 (2)
O1^ii^—Cu1—O3W	94.01 (6)	C11—C10—H10	120.1
O1—Cu1—O3W	85.99 (6)	C9—C10—H10	120.1
N1—Cu1—O3W	87.85 (7)	N2—C11—C10	121.8 (2)
N1^ii^—Cu1—O3W	92.15 (7)	N2—C11—H11	119.1
O3W^ii^—Cu1—O3W	180.00 (4)	C10—C11—H11	119.1
O5—Cu2—O5^iii^	180.0	N2—C12—C13	122.2 (2)
O5—Cu2—N2	83.06 (7)	N2—C12—C14	114.18 (19)
O5^iii^—Cu2—N2	96.94 (7)	C13—C12—C14	123.7 (2)
O5—Cu2—N2^iii^	96.95 (7)	C12—C13—C9	119.4 (2)
O5^iii^—Cu2—N2^iii^	83.05 (7)	C12—C13—H13	120.3
N2—Cu2—N2^iii^	179.998 (1)	C9—C13—H13	120.3
O5—Cu2—O4W^iii^	94.09 (7)	O6—C14—O5	124.6 (2)
O5^iii^—Cu2—O4W^iii^	85.91 (7)	O6—C14—C12	119.9 (2)
N2—Cu2—O4W^iii^	86.13 (7)	O5—C14—C12	115.49 (19)
N2^iii^—Cu2—O4W^iii^	93.87 (7)	Cd1—O1W—H1A	106.3 (16)
O5—Cu2—O4W	85.91 (7)	Cd1—O1W—H1B	114.8 (16)
O5^iii^—Cu2—O4W	94.09 (7)	H1A—O1W—H1B	110 (1)
N2—Cu2—O4W	93.87 (7)	Cd1—O2W—H2A	104.0 (16)
N2^iii^—Cu2—O4W	86.13 (7)	Cd1—O2W—H2B	112.0 (16)
O4W^iii^—Cu2—O4W	180.0	H2A—O2W—H2B	108 (1)
C7—N1—C2	119.16 (18)	H3A—O3W—H3B	112 (1)
C7—N1—Cu1	129.85 (15)	H4A—O4W—H4B	109 (3)
C2—N1—Cu1	110.76 (13)	H5A—O5W—H5B	108 (3)
C11—N2—C12	119.09 (19)	H6A—O6W—H6B	108 (1)
C11—N2—Cu2	128.85 (15)	H7A—O7W—H7B	110 (3)
C12—N2—Cu2	112.00 (15)	H31—N3—H32	112 (1)
C1—O1—Cu1	113.79 (13)	H31—N3—H33	110 (1)
C1—O2—Cd1	119.57 (14)	H32—N3—H33	108 (1)
C14—O5—Cu2	114.67 (15)	H31—N3—H34	111 (1)
O2—C1—O1	125.21 (19)	H32—N3—H34	108 (1)
O2—C1—C2	118.48 (18)	H33—N3—H34	108 (1)
O1—C1—C2	116.30 (18)		

Symmetry codes: (i) −*x*, −*y*, −*z*+1; (ii) −*x*+1, −*y*, −*z*+1; (iii) −*x*+1, −*y*+1, −*z*+2.

### Hydrogen-bond geometry (Å, °)

**Table:**

*D*—H···*A*	*D*—H	H···*A*	*D*···*A*	*D*—H···*A*
O1W—H1A···O7^iv^	0.94 (2)	1.80 (1)	2.739 (2)	171 (2)
O1W—H1B···O4^v^	0.95 (2)	1.82 (1)	2.769 (2)	177 (3)
O2W—H2A···O3^v^	0.95 (1)	1.72 (1)	2.657 (3)	168 (2)
O2W—H2B···O8^vi^	0.95 (1)	1.77 (1)	2.722 (2)	177 (2)
O3W—H3A···O6W^vii^	0.94 (1)	1.84 (1)	2.781 (3)	172 (3)
O3W—H3B···O7^vii^	0.94 (1)	1.84 (1)	2.776 (3)	172 (3)
O4W—H4A···O3W^viii^	0.95 (1)	1.89 (1)	2.827 (3)	169 (2)
O4W—H4B···O4^iv^	0.95 (1)	1.80 (1)	2.752 (3)	176 (2)
O5W—H5A···O4W^ix^	0.95 (1)	2.10 (1)	3.048 (3)	170 (3)
O5W—H5B···O3	0.96 (1)	1.93 (1)	2.882 (3)	171 (3)
O6W—H6A···O6	0.95 (1)	1.79 (1)	2.742 (3)	173 (3)
O6W—H6B···O2W^x^	0.96 (3)	2.14 (2)	2.991 (3)	147 (2)
O7W—H7A···O2	0.95 (3)	2.09 (3)	3.009 (3)	163 (3)
O7W—H7B···O5W	0.95 (3)	1.93 (3)	2.861 (3)	165 (4)
N3—H31···O7W^ii^	0.98 (2)	1.90 (2)	2.867 (3)	174 (2)
N3—H32···O8	0.99 (2)	1.92 (1)	2.886 (3)	164 (2)
N3—H33···O5W^xi^	0.99 (1)	2.53 (2)	3.208 (4)	126 (2)
N3—H33···O5^xii^	0.99 (1)	2.30 (2)	3.131 (3)	140 (2)
N3—H33···O6^xii^	0.99 (1)	2.19 (2)	2.888 (3)	126 (2)
N3—H34···O8^xiii^	0.99 (2)	2.34 (1)	3.277 (3)	157 (2)

Symmetry codes: (iv) −*x*+1, −*y*+1, −*z*+1; (v) *x*, *y*, *z*+1; (vi) *x*−1, *y*−1, *z*; (vii) *x*, *y*−1, *z*; (viii) *x*, *y*+1, *z*+1; (ix) *x*, *y*−1, *z*−1; (x) *x*, *y*+1, *z*; (ii) −*x*+1, −*y*, −*z*+1; (xi) *x*+1, *y*+1, *z*+1; (xii) *x*+1, *y*, *z*; (xiii) −*x*+2, −*y*+1, −*z*+1.

## References

[bb1] Bruker (2007). *SMART* and *SAINT*. Bruker AXS Inc., Madison, Wisconsin, USA.

[bb2] Caneschi, A., Gatteschi, D., Lalioti, N., Sangregorio, C., Sessoli, R., Venturi, G., Vindigni, A., Rettori, A., Pini, M. G. & Novak, M. A. (2001). *Angew. Chem. Int. Ed.* **40**, 1760–1763. 10.1002/1521-3773(20010504)40:9<1760::aid-anie17600>3.0.co;2-u 11353503

[bb3] Dong, Y.-B., Smith, M. D. & zur Loye, H.-C. (2000). *Angew. Chem. Int. Ed.* **39**, 4271–4273. 10.1002/1521-3773(20001201)39:23<4271::AID-ANIE4271>3.0.CO;2-1 29711887

[bb4] Evans, O. R. & Lin, W. (2002). *Acc. Chem. Res.* **35**, 511–522. 10.1021/ar0001012 12118990

[bb5] Kitagawa, S., Kitaura, R. & Noro, S. (2004). *Angew. Chem. Int. Ed.* **43**, 2334–2375. 10.1002/anie.200300610 15114565

[bb6] Kitagawa, S., Noro, S. & Nakamura, T. (2006). *Chem. Commun.* pp. 701–707. 10.1039/b511728c 16465313

[bb7] Kitagawa, H., Onodera, N., Sonoyama, T., Yamamoto, M., Fukawa, T., Mitani, T., Seto, M. & Maeda, Y. (1999). *J. Am. Chem. Soc.* **121**, 10068–10080.

[bb8] Li, Z.-G., Wang, G.-H., Jia, H.-Q., Hu, N.-H. & Xu, J.-W. (2008). *CrystEngComm*, **10**, 173–176.

[bb9] Noro, S., Kitagawa, S., Yamashita, M. & Wada, T. (2002*a*). *Chem. Commun.* pp. 222–223. 10.1039/b108695b 12132482

[bb10] Noro, S., Kitagawa, S., Yamashita, M. & Wada, T. (2002*b*). *CrystEngComm*, **4**, 162–164.

[bb11] Sheldrick, G. M. (1996). *SADABS*. University of Göttingen, Germany.

[bb12] Sheldrick, G. M. (2008). *Acta Cryst.* A**64**, 112–122. 10.1107/S0108767307043930 18156677

[bb13] Wang, G.-H., Li, Z.-G., Jia, H.-Q., Hu, N.-H. & Xu, J.-W. (2009). *Acta Cryst.* C**65**, m333–m336. 10.1107/S010827010902890X 19726845

